# Anti-psoriatic characteristics of ROCEN (topical Arthrocen) in comparison with Cyclosporine A in a murine model

**DOI:** 10.1186/s12906-024-04405-5

**Published:** 2024-02-24

**Authors:** Ramin Goudarzi, Min-Ho Kim, Alireza Partoazar

**Affiliations:** 1 Division of Research and Development, Pharmin USA, LLC, San Jose, CA USA; 2 Department of Biological Sciences, Kent State University, Kent, OH 44240 USA; 3 Experimental Medicine Research Center, Tehran University of Medical Sciences, Tehran, Iran

**Keywords:** Liposome, Arthrocen, Animal, Inflammation, Psoriasis

## Abstract

Topical ROCEN (Roc), liposomal arthrocen hydrogel, is a robust anti-inflammatory formulation which has been developed for skin diseases such as eczema. Therefore, we aimed to evaluate the efficacy of Roc 2% on the healing of imiquimod (Imiq)-induced psoriasis in a mouse model. Psoriasis was induced by applying Imiq topically to the mice's back skin once daily for five consecutive days. Moreover, a group of animal experiments was treated with Cyclosporine A (CsA), as a standard drug, for comparison with Roc treated group. The efficacy of Roc on skin lesions was evaluated by employing Psoriasis Area and Severity Index (PASI) scores. Subsequently, the skin samples were assessed using Baker’s scoring system and Masson's trichrome staining, immunohistochemistry, and real-time PCR analysis. The observational and histopathological results indicated that topical application of Roc significantly reduced the PASI and Baker’s scores in the plaque-type psoriasis model. Moreover, biochemical assessments showed that Roc suppressed significantly the increase of IL-17, IL-23, and TNF-α cytokines gene expression in the lesion site of psoriatic animals. In conclusion topical Roc 2% could significantly alleviate major pathological aspects of Imiq-induced psoriasis through inflammation inhibition which was comparable to the CsA drug. The beneficial outcomes of Roc application in the psoriasis model suggest its potential usage in complementary medicine.

## Introduction

Psoriasis is one of the most common immunological skin diseases that may be caused by hereditary, environmental, lifestyle factors and/or a combination of them [1]. Psoriasis is indexed by red plaques with white or silver scales on the red patches which frequently appear on the skin as localized or widespread. Disruption of keratinocyte proliferation and maturation causes acanthosis, hypogranulosis, parakeratinization, and proliferation of cutaneous blood vessels, which are hallmarks of psoriatic plaques [2]. However, the primary pathogenetic mechanism and the cell types involved in the onset of the disease are still under debate. It is mentioned that primary effector cells are dermal dendritic cells (DCs), in particular, plasmacytoid DCs (pDCs). Those activations will trigger inflammatory cytokines cascade including T-cell-derived lymphokines such as Interferon-γ (IFN-γ), tumor necrosis factor (TNF)-α, IL-17, and antigen-presenting cell cytokines such as IL-23 [3].

Psoriasis skin lesions elevate the levels of inflammatory cytokines, which can be linked to the severity of the disease and the amount of neovascularization in the skin as erythema [4]. It is known that inflammatory cytokines such as tumor necrosis TNF-α, IL-17, and IL-23 have a key role in the progress of psoriatic lesions. T helper cells of type 17 can generate elevated amounts of IL-17 upon inflammatory stimulation. This IL-17 then interacts with keratinocytes, causing an increase in the thickness of the epidermis which is known as epidermal hyperplasia [3].

Since IL-17, IL-23, and TNF-α cytokines certainly contribute to sustained inflammation within psoriatic disease, new treatment strategies are advised for the inflammation blockage in the lesion site. Topical medication is the main route of therapy in patients with mild disease of psoriasis lesions within the face, flexures, and nails. However, limitations in therapeutic efficacy in the target tissue and adverse effects of conventional drugs suggest research on the new therapeutics [5, 6]. For example, epidermal hyperplasia, hyperkeratosis, and reduced hydration of the skin restrict the drug permeation, consequently the therapeutic efficacy of conventional topical therapy. Moreover, cheilitis, alopecia, desquamation, drying of mucous membranes, pruritus, etc. are adverse effects of employing conventional topical drugs in psoriasis [6]. Therefore efforts should be made in direct to reduce the side effects of drugs like CsA through different approaches like complementary therapy with new components [7].

Arthrocen as the main material of Roc 2% gel, is an avocado/soybean unsaponifiables compound and is a dietary supplement FDA approved. Roc has been designed as a nanoliposomal topical formulation with analgesic [8], anti-inflammatory, and wound-healing properties [9, 10]. The evidence shows that both oral arthrocen and topical Roc have considerable potency in the suppression of inflammatory pathways in clinical [11] and animal studies [12, 13]. Arthrocen mediates anti-inflammatory effects by the reduction of the activity of MPO and TNF-α and inhibiting the expression of NF-kB protein, and IL-17 (as a signature cytokine of the Th17 subset) [11, 14].

Hence, we intended to study the anti-psoriatic aspects of the Roc in the animal model. The study aimed to assess the severity of skin inflammation concerning PASI scores in mice with Imiq-induced psoriasis. Additionally, the effect of topical Roc on the other psoriasis indices was evaluated using Baker’s scoring system and histopathological analysis, real-time PCR, and immunohistochemistry (IHC).

## Material & method

### Drugs and formulation

Cyclosporine A (CsA) powder and Arthrocen were obtained from Sigma (USA) and Pharmin USA, LLC (USA), respectively. Aldra 5% cream (Imiquimod) was obtained from Meda Pharmaceuticals (UK). Topical Roc was prepared as described in our previous studies [8]. The formulation was composed of a lipid mixture of soy lecithin and cholesterol (10:1) and arthrocen 2% in a gel form. The CsA solution was prepared by dissolving it in 10% ethanol at a concentration of 10 mg/mL.

### Animals

BALB/c male mice with an average weight of 30 g were prepared from Tehran University of Medical Sciences. Only animals with healthy skin (without erythema or scratches) were included in the experiments. They had free access to water and food and had 12 h of light and 12 h of darkness at a temperature of 21 ± 2 degrees Celsius during the study. The experimental methods were deeply in line with the experiment and ethics followed the guidelines set out by the Canadian Council for Animal Care and approved by the institutional Animal Care and Use Committee, No: 1402.328.

### Experimental design and psoriasis induction

We designed our experiment to consist of six mice for each group as follows: *VEH* group: Vehicle alone; *Imiq* group: Aldra + vehicle as the negative control; *Imiq*-*CsA* group: Aldra + cyclosporine as a positive treatment control; *Imiq-ROC* group: Aldra + ROCEN. First, mice received topically Imiq cream 5% at the dose of 62.5 mg on the shaved back to induce psoriasis-like inflammation for five consecutive days [5]. Morover, 2 h after exposure to Imiq, back skin of mice were topically treated with 0.2 mL Roc 2%, CsA, or vehicle in relative groups during 5 days. For the VEH group, mice were treated with the vehicle without Imiq. In addition, all animals were weighed regularly once a day, and at the end of experiments, mice were euthanized by CO2, and then their back skin and spleen tissues were harvested for further analysis.

### PASI score evaluation

The severity of skin inflammation was indicated daily by PASI score during the treatment. The objective scoring system was indexed by the erythema, scaling, and thickening with the score range from zero to four (zero: none; one: slight; two: moderate; three: marked; four: very marked). Then, all the scores were summed up as the cumulative score (scale 0–12) to indicate the severity of inflammation [15]. The evaluation was done independently by two researchers (*n* = 6 for each parameter) and the mean of values was then calculated.

### Histopathological examinations

After euthanizing mice, skin samples were collected and then fixed in 10% v/v formaldehyde diluted in phosphate buffer saline, followed by paraffin embedding. A blinded inspector evaluated Hematoxylin–eosin-stained skin Sects. (3- 4 μm) for the histological parameters characteristic of psoriasis using Baker’s scoring system. We graded the histopathological score on a scale from 0 to 11 [16]. Also, prepared samples were stained with Masson’s Trichrome staining to evaluate the detailed histological features of tissues. Briefly, the skin samples were deparaffinized and stained using the hematoxylin solution, a mixture of orange-G, ponceau acid fuchsin, and a light green coloring agent. The epidermal thickness values were measured using ImageJ software through length measurements.

### IHC analysis

The immunohistochemistry (IHC) method was utilized to evaluate the expression of TNF-α cytokine in the back skin of treated mice. Briefly, specimens fixed in buffered formalin (10% v/v) were dehydrated and embedded in paraffin. The procedure of primary antibodies and specific secondary antibodies applied in this study was conducted according to our previous study [17] and was analyzed blindly by an expert pathologist. The existence of brown cells in the stained sections represented the expression of the relative tissue cytokine.

### Quantification by real-time PCR

For each mouse, a proper skin sample was also harvested sterilely and immediately preserved as flash-frozen in liquid nitrogen and then stored at -80 °C. The mRNA expressions of IL-17, IL-23, and TNF-α factors were measured quantitatively by real-time PCR analysis. Total RNA was isolated from all tissues using an RNA X-Plus kit (Cinna Gen, Tehran, Iran) and was quantified by NanoDrop spectrophotometer (Thermo Scientific Nanodrop 2000). In the next step, cDNA was synthesized (Bio fact, South Korea), and real-time PCR was performed with the Cyber Green method (Yekta Tajhiz Co, Cat No: YT2551) with the following program, 40 cycles of denaturation (15 s at 95 °C), annealing (20 s at 60 °C) and elongation process (30 s at 72 °C). β-actin was used as the housekeeping gene and positive control. The relative expression of transcript levels of each case was calculated according to 2 ^–ΔΔct^ [5].

### Statistical analysis

We analyzed data and drew the charts by GraphPad Prism 8. All data are presented as mean ± standard error of the mean (SEM) and were analyzed by one-way analysis of variance (ANOVA). *P* < *0.05* was considered significant.

## Results

### Observational scores in psoriasis inflammation

**Fig. 1:**
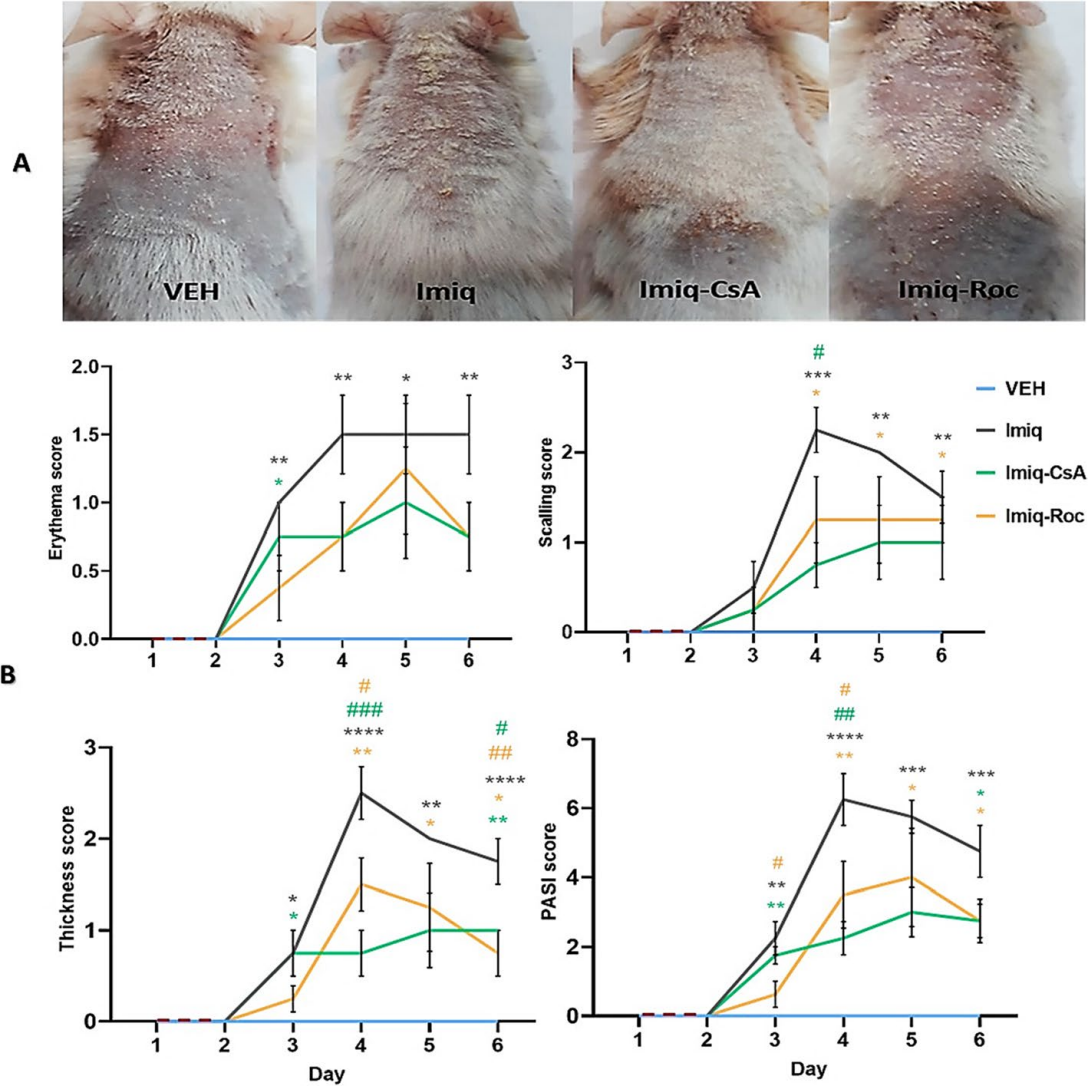
Topical application of Roc 2% attenuates Imiq-induced inflammation like psoriasis in mice. **A** The representative mice back skins on day 6 of experiments showed signs of erythema, scaling, and thickening that appeared obviously in untreated psoriatic mice while relative signs were improved in treatment groups at the end of the experiment. **B** Erythema, Scaling, Thickness, and PASI scores on a scale from 0 to 4 for 6 consecutive days. Data were indicated by the mean ± SEM and *n* = 6 per group. Green, brown, and dark colors are related to Imiq-CsA, Imiq-Roc, and Imiq groups, respectively. * *P* < *0.05*, ** *P* < *0.01*, *** *P* < *0.001*, and **** *P* < *0.0001* were compared to the VEH group. ^#^ *P* < *0.05*, ^##^ *P* < *0.01*, and ^###^ *P* < *0.001* were compared to the Imiq group

Both Roc and CsA revealed a noticeable improvement in inflammatory signs including erythema, scaling, and thickening in mice skin subjected to Imiq (Fig. 1A). While inflammatory signs appeared in the early stage of Imiq application on the shaved back skin which continually increased severity of injury until the end of the experiment. Furthermore, changes in erythema, scaling, skin thickness scores as well as the cumulative PASI score were decreased considerably in the Imiq-Roc and Imiq-CsA groups in comparison with the Imiq group (Fig. 1B). Comparison between the Imiq group at time intervals of the experiment showed a significant (*P* < *0.05*) increase in inflammatory skin symptoms as well as cumulative PASI score compared with VEH group. Data with more details at different time points have been demonstrated in Fig. 1B.

### Histopathological markers

**Fig. 2:**
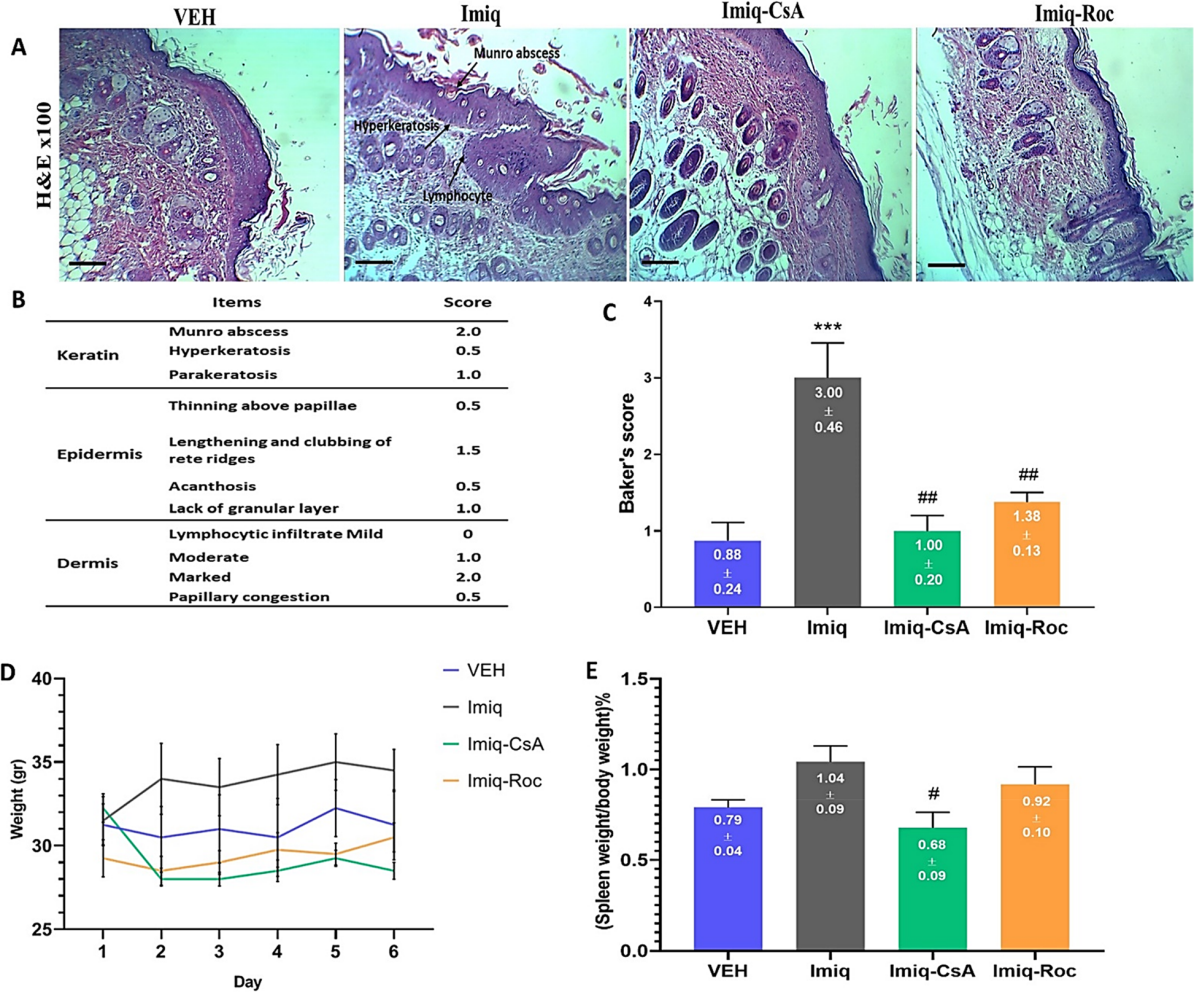
Illustration of histopathological analysis and spleen weight changes after applying Roc and CsA on psoriatic mice. **A** The representative histopathology of back skin stained with H&E showed Imiq group has Munro’s microabscesses in the keratin layer, lengthening and clubbing of rete ridges, and moderate-severe dermal lymphocytic infiltrate while Roc 2% was similar to CsA which reversed the Imiq-induces pathologic changes in mice receiving Imiq. **B** The indications of Baker’s scoring system [15] reflected in the method section were used for histopathology evaluation. **C** Bars represent Baker’s pathology score after 5 days of applying different treatments with Imiq on the shaved back skin of mice. **D** Mice’s weight was measured daily for up to 6 days. **E** A significant decrease in the ratio percentage of the spleen mass to the weight of mice was seen in the Imiq-CsA treatment group. Symbols on the columns indicate the mean ± SEM with *n* = 6 per group. *** *P* < *0.001* were compared to the VEH group. ^#^ *P* < *0.05*, ^##^ *P* < *0.01* as compared to the Imiq group

The histopathologic analysis was carried out by H&E staining and using Baker’s scores as given in Fig. 2A and B, respectively. Data in Fig. 2C indicate that Imiq treatment caused a significant *(P* < *0.05)* psoriatic-like reaction compared with the VEH group. Regarding pathological assessments, both CsA and Roc treatments showed statistically a significant (*P* < *0.05*) difference in Baker’s score in the reduction of inflammation compared with the Imiq group. Moreover, our results in Fig. 2D display an independent impact on changes in mice’s weight regarding time and treatment. Therefore, there was no significant, *P* > *0.05*, difference between the groups in the study. As shown in Fig. 2E, spleen enlargement was measured following Imiq treatment, to determine the percentage of spleen weight/ body weight. Although the Imiq-CsA group only has a significant (*P* < *0.05)* ratio percentage reduction of spleen weight/ body in comparison to the Imiq group. Imiq-Roc treatment could also considerably decrease this ratio number in inflamed mice.

### Epidermal thickness and hyperkeratosis

**Fig. 3:**
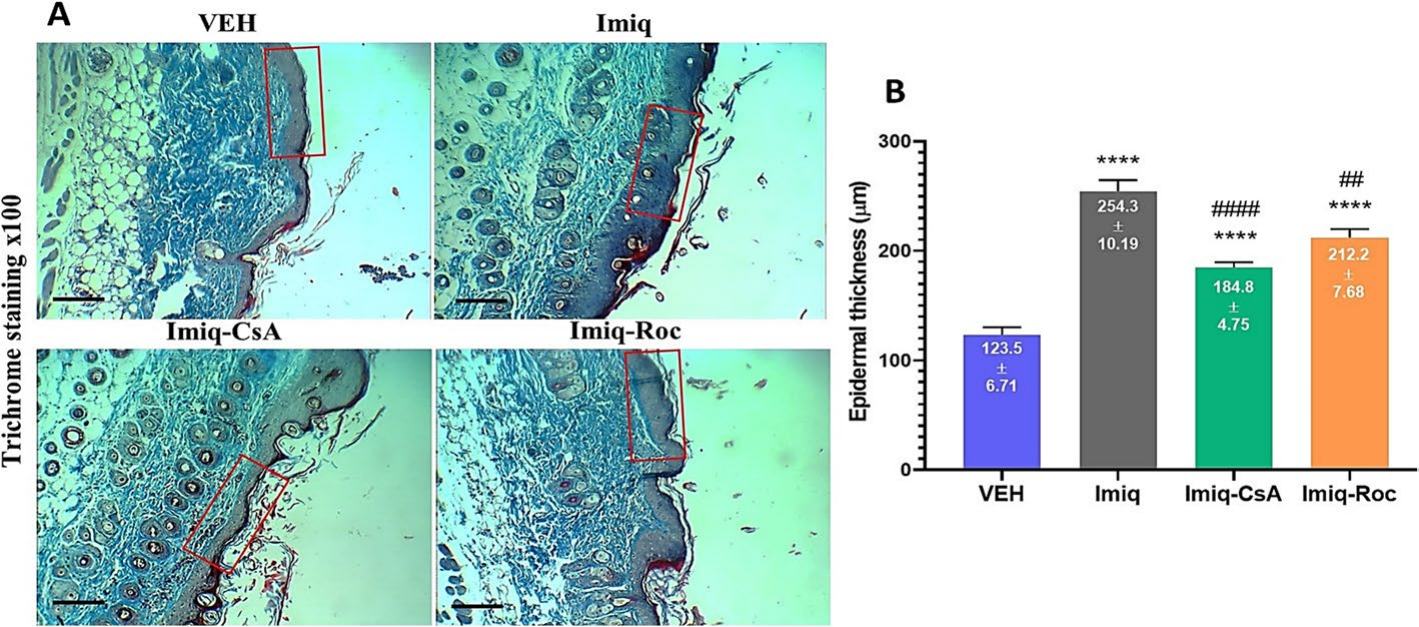
Histological examination by Masson’s trichrome staining of back skin of relative groups. **A** Roc treatment evaluation showed a decrease in the epidermal thickness which was assessed for each experiment based on the four smooth regions enclosed by red rectangular. **B** Epidermal thickness values were measured using Image J at day 6. Symbols on the columns indicate the mean ± SEM with *n* = 6. ^****^ P < *0.0001* was compared to the VEH group. ^*##*^* P* < *0.01* and ^*####*^* P* < *0.0001* were compared to the Imiq group

The histological observation of Masson’s trichrome staining indicated that Imiq significantly *(P* < *0.05)* increased thickness measurement and hyperkeratosis in the epidermal region of dorsal skin compared to VEH (Fig. 3A). According to Figs. 3A and B, both Imiq-CsA and Imiq-Roc decreased the epidermal thickness and keratosis in macroscopic fields when are compared with VEH group. Moreover, Imiq-CsA was shown more potent than Imiq-Roc in the improvement of Imiq-induced psoriasis however, their values of thickness analysis were equally significant, *P* < *0.05*, compared to the Imiq group (Fig. 3B).

### TNF-α in skin subjected to Imiq

**Fig. 4:**
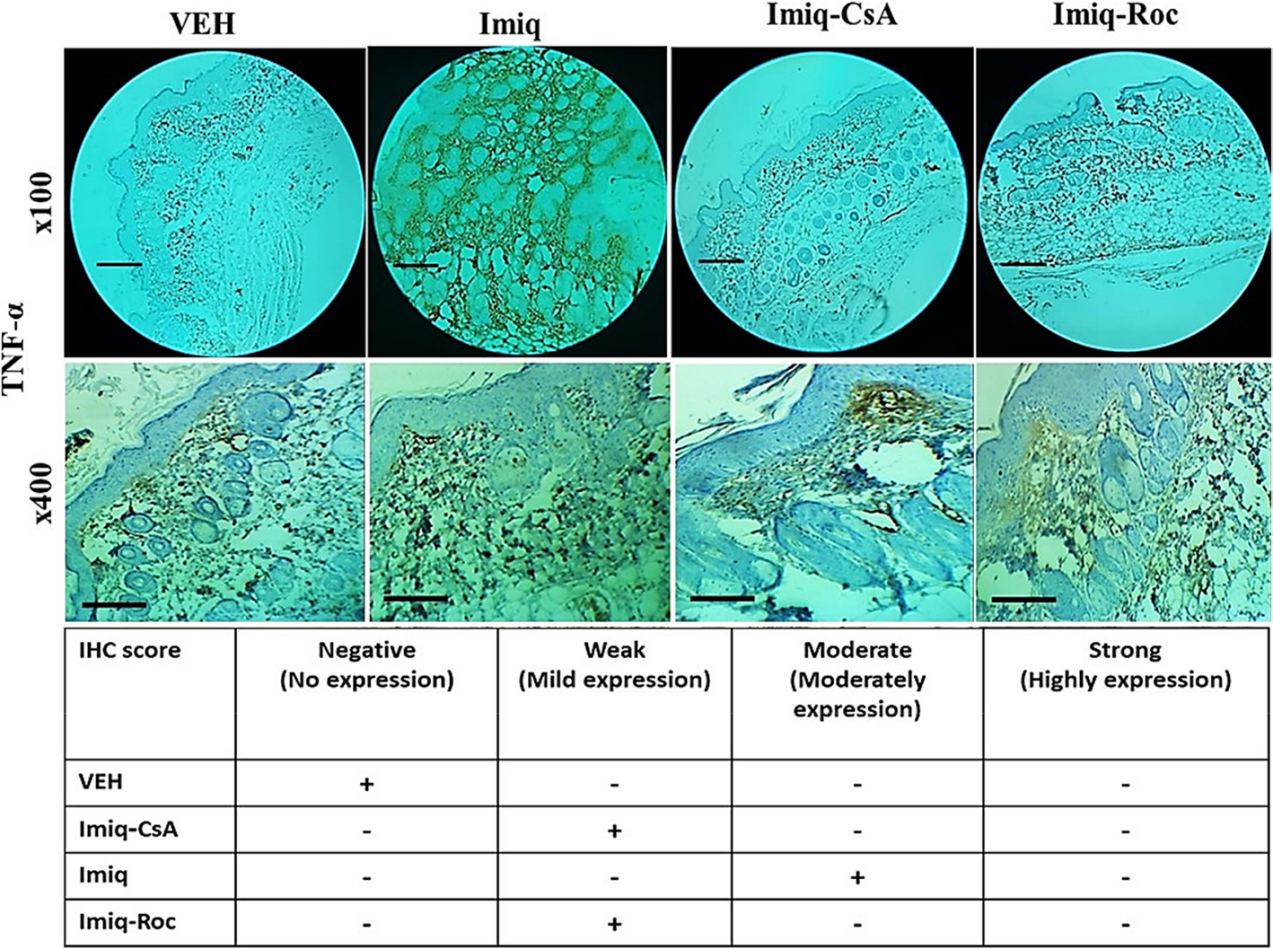
Immunohistochemical analysis in back skin samples of mice presents brown cells as the expression of the TNF-α cytokine markers in different magnifications. The indication scores show that Roc and CsA treatments reduced the tissues’ TNF-α marker in mice receiving Imiq

In this study, immunohistochemical analysis of TNF-α was performed in back skin mice samples. The cytokine rate in the sections was interpreted following scores of negative (no expression), weak (mild expression), moderate (moderately expression); and severe (highly expression). The analysis showed moderately expression in the dermis on the 6th day of the Imiq group compared with the VEH group, as given in Fig. 4. The TNF-α indices were at level weak for both Roc and CsA treatment groups as observed in IHC staining.

### Inflammatory gene expression

**Fig. 5:**
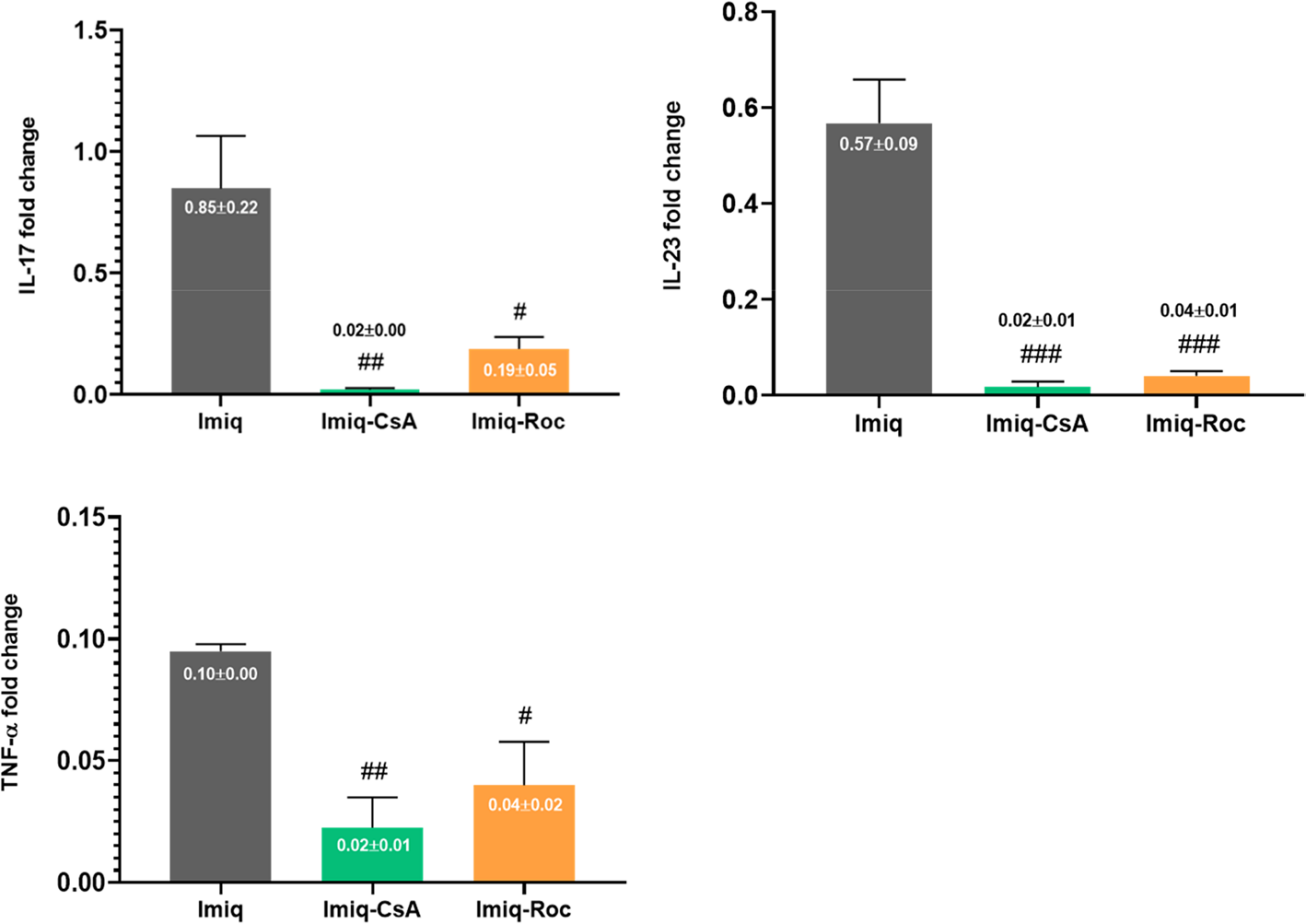
The representative gene expression cytokines using real-time PCR in the cutaneous samples of mice subjected to Imiq. Topical Roc and CsA reduced significantly the Imiq-induced cutaneous expression of IL-17, IL-23, and TNF-α. The data are expressed as mean ± SEM of three mice per group. ^#^ *P* < *0.05*, ^##^ *P* < *0.01*, and ^###^ *P* < *0.001* were compared to the Imiq group

In this research, we determined the expression of key mediators of IL-17, IL-23, and TNF-α which are involved in the pathogenesis of psoriasis-like inflammation by the RT-PCR technique. The expression of all the cytokines exhibited a significantly (*P* < *0.05*) lower level in Imiq-CsA in comparison with the Imiq group (Fig. 5). We chose CsA in all experiments as a standard control drug for better judgment about the potency of Roc formulation in the suppression of psoriasis due to inflammation. Moreover, Roc potentially, *P* < *0.05*, inhibited gene expression of IL-17, IL-23, and TNF-α levels that those values associated with cytokines expression reduction were near in Imiq-CsA (Fig. 5).

## Discussion

In this study, a psoriasis model was induced by Imiq in mice which simulates psoriatic inflammation in humans [18]. Our data showed Roc improved erythematous plaques, overlying scale, increasing skin thickness, and hyperkeratosis appeared in Imiq-induced psoriasis in mice. The histopathological analysis also showed the reduction of inflammation which was indicated by Baker’s score in the Roc treating group. Moreover, Roc inhibited significantly inflammatory cytokines gene expression of IL-17, IL-23, and TNF-α in psoriatic lesions.

In parallel with the Roc application, we used CsA as the standard treatment of severe inflamed/ immune-mediated skin disorders such as psoriasis and atopic dermatitis in the clinic [19]. Interestingly, our findings revealed that treatment with Roc similar to the CsA drug diminished the levels of gene expression of relative inflammatory cytokines in the experiments. The challenges due to possible side effects, relapse of disease after treatment, and poor absorption of CsA through the skin are limitations [20] which justify the research and development of new formulations like Roc. Following topical CsA application, some complications such as mild erythema, dryness, and fissuring can appear on the skin [21].

Furthermore, our results showed a significant decrease in spleen enlargement following 5-day CsA treatment in psoriatic animals; however, further immunologic analysis on spleen samples is needed to determine systemic inflammation. Topical Roc has been designed for the alleviation of local inflamed injuries such as burn wound, pain, atopic dermatitis, or other inflammatory skin diseases in our lab [8–10]. Roc is a biocompatible liposomal formulation which possesses high efficiency in the healing of skin injuries [22, 23]. The main active compound of Roc is arthrocen, an FDA approved supplementary drug, that has been used orally in osteoarthritis therapy and control of pain in the clinic [11]. According to our findings, one of the main mechanisms of Roc in control of Imiq-induced psoriasis can be via its robust anti-inflammatory effect in the suppression of IL-17, IL-23, and TNF-α expression associated with skin inflammation.

It has been proposed that the IL-23/IL-17 axis has a key role in psoriasis promotion. Skin-resident dendritic cells DCs and macrophages rather than migratory myeloid cells are the primary sources of IL-23 [3, 24]. Also, activated pDCs in the dermis secrete type I interferon and TNF-α. These DCs will produce IL-12 and IL-23, and then lead immature T cells into T helper (Th)1, Th17, and Th22 cells. These pro-inflammatory cytokines contribute to the proliferation of epithelial cells and hyperkeratosis, and the involvement of more inflammatory immune cells, resulting in psoriatic lesions [25]. Therefore, the blockage of TNF-α followed by monoclonal antibodies which leads to suppressing IL-17 and IL-23, has been noticed in alleviating psoriatic severity in the clinic [25–27].

Clinical and experimental evidence recommended that arthrocen, the main compound of Roc, has a potential inhibitory effect on inflammatory cytokines production like TNF-α and IL-17 which has been in line with our data. Recently, a randomized double-blind placebo-clinical trial by arthrocen showed that its oral administration diminished TNF-α and IL-17 while it increased anti-inflammatory IL-4 and IL-10 blood levels in patients with osteoarthritis [11]. Also, in an experimental study, Roc decreased significantly IL-8 and TNF-α cytokines production in the dorsal skin of mice subjected to atopic dermatitis [9]. Atopic dermatitis is mainly caused by immune stimuli and is characterized by severe itching, a mild to severe rash, edema, hemorrhage, and erosion of the skin surface [28]. The study by Goudarzi et al. [9] showed that Roc alleviates skin tissue damage, pruritus, and inflammatory cytokines expression associated with a contact stimulus in atopic dermatitis.

In conclusion, topical administration of Roc in psoriatic mice could reduce dermatitis symptoms and inflammatory cytokines of IL-17, IL-23 & TNF-α, which was comparable to the CsA drug in our experiment. The safety and effectiveness of topical Roc in the control of psoriasis justify its application in clinical trials. However, additional investigation will be desirable for more understanding of more mechanisms involved in Roc affecting psoriasis.

## Data Availability

The datasets analyzed during the current study are available from the corresponding author upon reasonable request.
